# Cross-Link Density, Mechanical and Thermal Properties of Chloroprene Rubber Cross-Linked with Silver(I) Oxide

**DOI:** 10.3390/ma15062006

**Published:** 2022-03-08

**Authors:** Piotr Kobędza, Aleksandra Smejda-Krzewicka, Krzysztof Strzelec

**Affiliations:** Institute of Polymer and Dye Technology, Faculty of Chemistry, Lodz University of Technology, Stefanowskiego 16, 90-537 Łódź, Poland; aleksandra.smejda-krzewicka@p.lodz.pl (A.S.-K.); krzysztof.strzelec@p.lodz.pl (K.S.)

**Keywords:** silver(I) oxide, chloroprene rubber, cross-linking, cross-link density, DSC, TGA, tensile properties, elasticity constants

## Abstract

The purpose of this work was to cross-link chloroprene rubber (CR) with silver(I) oxide (Ag_2_O) and to investigate the properties of the obtained vulcanizates. Silver(I) oxide was chosen as an alternative to zinc oxide (ZnO), which is part of the standard CR cross-linking system. The obtained results show that it is possible to cross-link chloroprene rubber with silver(I) oxide. This is evidenced by the determined vulcametric parameters, equilibrium swelling and elasticity constants. As the Ag_2_O content in the composition increases, the cross-link density of the vulcanizates also increases. However, the use of 1 phr of Ag_2_O is insufficient to obtain a suitably extensive network. Exclusively, the incorporation of 2 phr of Ag_2_O results in obtaining vulcanizates with great cross-link density. The obtained compositions are characterized by good mechanical properties, as evidenced by high tensile strength. The performed thermal analyses—differential scanning calorimetry (DSC) and thermogravimetry (TGA) allowed us to determine the course of composition cross-linking, but also to determine changes in their properties during heating. The results of the thermal analysis confirmed that CR can be cross-linked with Ag_2_O, and the increasing amount of oxide in the composition increases the degree of cross-linking of vulcanizates. However, the amount of Ag_2_O in the composition does not affect the processes occurring in the heated vulcanizate.

## 1. Introduction

Chloroprene rubber (CR) is a specialist elastomer, characterized by good mechanical properties, good adhesion, resistance to technical media, crystallization ability and thermo-cross-linking ability [1,2,3]. The most important parameter that characterizes CR is increased flame resistance [4,5,6]. Chloroprene rubber is normally cross-linked with zinc oxide in the presence of magnesium oxide. However, CR can also be cross-linked with other substances. A phenol-formaldehyde resin can be used as a cross-linking agent [7]. CR cross-linking is also possible with carbon nanodots. In addition to the function of the cross-linking agent, nitrogen-doped carbon nanodots can also be used as reinforcing fillers [8]. It is interesting to use liquid polysulfide polymer as a cross-linking agent in thermally initiated thiol-ene reaction. The obtained material is self-healing, recyclable and re-cross-linkable [9]. A material with similar self-healing and re-cross-linkable properties can be obtained by using a latent catalyst in the form of an organic copper (II) methacrylate complex. Zinc oxide and sulfur are used for cross-linking of such a composition containing an organic copper (II) methacrylate complex [10]. Chloroprene rubber, but also bromobutyl rubber (BIIR), can be cross-linked with iron (III) acetylacetonate in the presence of triethanolamine [11]. CR can also be radiation cross-linked. This cross-linking requires the presence of diallyl ester of maleic acid or an epoxy resin in the composition, as well as zinc oxide [12]. Chloroprene rubber is often used in blends with other rubbers, for example, butadiene rubber (BR) or styrene-butadiene rubber (SBR). In such materials, metal oxides can be used for the cross-linking: copper oxides [13,14,15] or iron oxides [16,17]. In the case of blends of chloroprene rubber with natural rubber (NR), ethylene thiourea as accelerator can be replaced by an alkanolamide [18]. In the case of CR and BIIR blends, low-temperature cross-linking (at 90 °C) is possible. Chlorinated polyethylene was used as a compatibilizer, thiocarbanilide or modified di-o-tolyl guanidine were used as accelerators and zinc oxide was used as a cross-linking agent [19].

Silver(I) oxide (Ag_2_O) is in the form of black or black-brown powder with a cubic crystal system, very slightly soluble in water [20,21]. The preparation of Ag_2_O from the elements is difficult and requires the use of high pressure. Typically, it is obtained by addition of alkali hydroxide (e.g., NaOH) to a solution of a silver(I) salt, e.g., AgNO_3_ [22,23]. It is also possible to oxidize atomic silver, but it requires specific conditions, and only a thin layer of silver oxide is produced [24]. Ag_2_O is often used to convert chlorides of other metals into hydroxides. Reaction with hydrogen, carbon monoxide and organic compounds results in the reduction of Ag_2_O to metallic silver. Silver(I) oxide is used in the Tollens’ test, where it is one of the main reactants [21]. The most important feature that determines the use of silver and its derivatives, including silver(I) oxide, is its antibacterial properties. They are the result of the ability of silver ions to inactivate many bacterial enzymes and bind to nucleic acids, which are the genetic material of microorganisms [25]. In the case of silver oxide, it can be used as an antibacterial coating material for cotton fibers [26]. However, Ag_2_O often finds application in other fields. In organic chemistry, silver(I) oxide is used as a mild oxidizing agent. For example, it oxidizes aldehydes to carboxylic acids [27,28]. Due to the conductive properties of silver(I) oxide, which is classified as a p-type semiconductor, it is of interest to the electronics industry [29,30]. It can be used as a substitute for silver paste in low-temperature-cured metallo-organic decompositions. These materials are used to produce radio-frequency identification tags [31]. Silver(I) oxide can be modified with graphene to increase the efficiency of Ag_2_O in electronics applications [32]. Ag_2_O in the form of thin films can be used in photovoltaic panels [30]. In addition, silver(I) oxide can be found in sensors, optical memories, batteries or capacitors [28,29,30,31,32]. In the case of polymers, silver(I) oxide can be used as a reinforcing filler to epoxy resin, causing an increase in hardness and compression strength and a reduction in wear rate [33].

The purpose of this work was to cross-link chloroprene rubber (CR) with silver(I) oxide (Ag_2_O) and to investigate the properties of the obtained vulcanizates. This is due to the need to find an alternative to ZnO. European Union regulations force a significant limitation of the use of ZnO in various fields, including elastomer technology. The regulations result from the harmful effects of ZnO on aquatic organisms. As an alternative to CR cross-linking agent, other metal oxides such as copper oxides [13,14,15] or iron oxides [16,17] can be used. Ag_2_O as a CR cross-linking agent has not been described in the literature so far, which made the following results possible as presented here.

## 2. Experimental Part

### 2.1. Materials

In this study, chloroprene rubber (CR) (Baypren^®^216 MV from Lanxess GmbH, Cologne, Germany), with a density of 1.23 g/cm^3^ and Mooney viscosity (ML 1 + 4 100 °C) of 43 ± 5 was used. Silver(I) oxide (Ag_2_O) (abcr GmbH, Karlsruhe, Germany) with a density of 7.14 g/cm^3^, purity of 99%, specific surface area of 0.382 m^2^/g and particle size of 270 nm was used as the cross-linking agent. Stearic acid (Chemical Worldwide Business Sp. z o.o., Słupca, Poland) with a density of 0.85 g/cm^3^ was used as the dispersing agent.

### 2.2. Research Methods

The chloroprene rubber composites were prepared using a Krupp-Gruson laboratory two-roll mill (Laborwalzwerk 200 × 450, Krupp-Gruson, Magdeburg-Buckau, Germany) with a roll diameter of 200 mm and 450 mm long. The temperature of the roll was 20–25 °C, while the speed of the front roll was 20 rpm, with the roll’s friction 1:1.25. The preparation of the samples was conditioned for 24 h.

Vulcametric measurements were determined by the Alpha Technologies MDR 2000 rotorless rheometer (MDR 2000, Alpha Technologies, Hudson, OH, USA), heated to 160 °C. The oscillation frequency was 1.67 Hz. The test was 60 min and was performed according to ASTM D5289. The torque increment after the specified time of heating was calculated from Formula (1):

(1) $$∆M_{x}=M_{x}-M_{\min}$$

where: Δ*M*_x_ is the torque increment after the specified time of heating (dN·m), *M*_x_ is the torque after the specified time (dN·m) and *M*_min_ is the minimum torque (dN·m).

The cure rate index (CRI) was calculated from Formula (2):

(2) $$\mathrm{CRI}=\frac{100}{t_{90}-t_{02}}$$

where: *t*_90_ is the vulcanization time (min), and *t*_02_ is the scorch time (min).

Vulcanization was performed in an electrically heated hydraulic press. Appropriate amounts of the compositions were placed in the steel molds, which were placed in a press at a temperature of 160 °C and a pressure of 200 bar. The vulcanization time was 30 min.

The determination of equilibrium volume swelling was performed. Samples were cut from the prepared vulcanizates in four different shapes. Each of them weighed from 25 to 50 mg, with an accuracy of 0.1 mg. Then, the samples were placed with solvents, toluene and heptane, in a weighing bottle. Prepared samples were placed in a thermostatic chamber for 72 h at 25 ± 1 °C, which after this time were bathed with diethyl ether, dried on filter paper and then weighed again. Then, the samples were dried in a dryer at the temperature of 50 °C for 24 h to a constant weight and they were reweighed. The equilibrium volume swelling (*Q_V_*) was calculated from Formula (3):

(3) $$Q_{V}=Q_{W}\cdot \frac{d_{v}}{d_{s}}$$

where: *Q_W_* is the equilibrium weight swelling (mg/mg), *d*_v_ is the vulcanizate density (g/cm^3^) and *d*_s_ is solvent density (g/cm^3^).

The equilibrium weight swelling was calculated from Formula (4):

(4) $$Q_{W}=\frac{m_{s}-m_{d}}{m_{d}^{*}}$$

where: *m*_s_ is the swollen sample weight (mg), *m*_d_ is the dry sample weight (mg) and *m*_d_^*^ is the reduced sample weight (mg).

The reduced sample weight was calculated from Formula (5):

(5) $$m_{d}^{*}=m_{d}-m_{0}\cdot \frac{m_{m}}{m_{t}}$$

where: *m*_0_ is the initial sample weight (mg), *m*_m_ is the mineral content in the blend (mg) and *m*_t_ is the total weight of the blend (mg).

The negative equilibrium weight swelling (*−Q_W_*), interpreted as the amount of leaching substances, was calculated from Formula (6):

(6) $$-Q_{W}=\frac{m_{0}-m_{d}^{*}}{m_{0}}$$

The rubber volume fraction (*V_r_*) was calculated from Formula (7):

(7) $$V_{r}=\frac{1}{1+Q_{V}}$$

The degree of cross-linking (*α_c_*) was calculated from Formula (8):

(8) $$\alpha_{c}=\frac{1}{Q_{V}}$$

The cross-link density (*ν_e_*) was calculated using Flory-Rehner Equation (9):

(9) νe=−Vr+μ·Vr2+ln1−VrV0·Vr13−Vr2

where: *µ* is the Flory-Huggins polymer-solvent interaction parameter (0.1329 for chloroprene rubber-toluene interaction [34]), and *V*_0_ is the molar volume of solvent (106.86 cm^3^/mol for toluene).

Determination of Mooney-Rivlin elasticity constants (*C*_1_, *C*_2_) was performed. The elasticity constants were calculated based on Mooney–Rivlin Equation (10):

(10) $$\frac{P}{2A_{0}\cdot \left(\lambda -\lambda^{-2}\right)}=C_{1}+C_{2}\cdot \lambda^{-1}$$

where: *P* is the deformation force at *λ* (kG), λ is the deformation (*λ* = *l*/*l*_0_), *l* is the measuring section of the sample loaded with *P* (cm), l_0_ is the measuring section of the unloaded sample (cm), *A*_0_ is the cross-sectional area of the unloaded sample (cm^2^), *C*_1_ is the first elasticity constant (kG/cm^2^) and *C*_2_ is the second elasticity constant (kG/cm^2^).

Extraction of vulcanizates in the boiling acetone vapors in a Soxhlet apparatus for 48 h was performed. After the given time, the samples were dried to a constant weight in a vacuum oven at 50 °C for 72 h. Results of the extraction allowed us to determine the content of non-rubber substances. The value of real extract (*E*_R_) was calculated from Formula (11):

(11) $$E_{R}=\frac{m_{0}-m}{m_{0}}$$

where: *E*_R_ is the real extract (mg/mg), *m*_0_ is the initial sample weight (mg) and *m* is the final sample weight (mg).

Mechanical properties: stress at elongation 100%, 200% and 300% (*S*_e100_, *S*_e200_ and *S*_e300_), tensile strength (*TS*_b_) and elongation at break (*E*_b_) were tested by the universal testing machine ZwickRoell 1435 (ZwickRoell, Ulm, Germany). The tests were performed according to PN-ISO 37:2007.

Thermal analyses—TGA and DSC—were performed using a Mettler Toledo TGA/DSC1 device (Mettler-Toledo, Columbus, Ohio, USA). TGA analyses were performed using a two-step procedure. First, samples of vulcanizates were heated in the temperature range of 25–600 °C in an argon atmosphere (flow rate 50 mL/min), with a heating rate of 20 °C/min. Next, the gas was changed into the air (flow rate 50 mL/min) and the heating was continued up to 900 °C with the same heating rate. DSC measurements were performed on rubber mixtures. Samples were heated from −100 °C to 250 °C, with a heating rate of 10 °C/min. Nitrogen (80 mL/min) was used as the protective gas, whereas liquid nitrogen was applied to cool the sample before the measurement.

## 3. Results and Discussion

To investigate the ability of chloroprene rubber cross-linking with silver(I) oxide, compositions containing 1, 2, 2.5, 3, 4 or 5 weight parts of Ag_2_O/100 weight parts of CR (phr) were prepared (Table 1). The purpose of silver(I) oxide use is to obtain vulcanizates with better properties compared to the materials obtained with the use of a standard cross-linking system. In addition, the use of zinc oxide is limited due to its harmful effect on aquatic organisms, and alternatives should be sought.

### 3.1. Vulcametric Parameters of CR Compositions Containing Silver(I) Oxide

**Figure 1:**
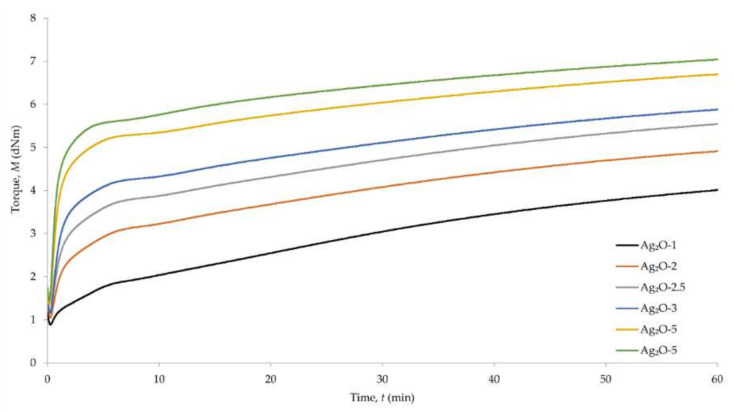
Vulcametric kinetics of chloroprene rubber containing silver(I) oxide (1–5 phr of Ag_2_O).

To determine the possibility of cross-linking chloroprene rubber with silver(I) oxide and the characteristics of the vulcanization course, vulcametric parameters were determined. The scorch time (*t*_02_) of the tested compositions was in the range from 0.45 min (for the composition containing 5 phr of Ag_2_O) to 6.93 min (for the composition containing 1 phr of Ag_2_O) (Table 2, Figure 1).

The scorch time value for the sample containing 1 phr of Ag_2_O differs significantly from the results for other samples. The incorporation of 2 phr of Ag_2_O results in the reduction of the scorch time to 1.18 min. The results of the vulcanization time (*t*_90_) show that the *t*_90_ decreases as the silver(I) oxide content in the composition increases. The longest vulcanization time was obtained for the sample containing 1 phr of Ag_2_O (*t*_90_ = 53.36 min), and the shortest vulcanization time was obtained for the sample containing 5 phr of Ag_2_O (*t*_90_ = 31.55 min). The value of the minimum torque (*M*_min_) increases with increasing Ag_2_O content in the sample. The smallest value was obtained for the sample containing 1 phr of Ag_2_O (*M*_min_ = 0.89 dN⋅m), and the highest value was obtained for the sample containing 5 phr of Ag_2_O (*M*_min_ = 1.45 dN⋅m). In addition, in the case of the torque increment value after 30 min of heating (Δ*M*_30_), the amount of silver(I) oxide affects the results obtained. The smallest torque increment was obtained for the sample containing 1 phr of Ag_2_O (Δ*M*_30_ = 2.16 dN⋅ m), and the largest torque increment was obtained for the sample containing 5 phr of Ag_2_O (Δ*M*_30_ = 4.99 dN⋅m). The values of the cure rate index (CRI) increase with increasing Ag_2_O content in the composition. The lowest value of the cure rate index was obtained for the composition containing 1 phr of Ag_2_O (CRI = 2.15 min^−1^), and the highest value was obtained for the composition containing 5 phr of Ag_2_O (CRI = 3.22 min^−1^).

The obtained results show a relationship between the amount of used silver(I) oxide and the vulcametric parameters. The greater amount of Ag_2_O results in a reduction of the scorch time and the vulcanization time, as well as an increase in the minimum torque, the torque increment after 30 min of heating and cure rate index. It is worth noting that the use of at least 2 phr of Ag_2_O results in noticeably shortening both the scorch time and vulcanization time. The differences between the results for samples containing higher amounts of silver(I) oxide are less than between the results when using 1 and 2 phr of Ag_2_O (5.75 min at *t*_02_ and 9.81 min at *t*_90_). A similar relationship is not visible for the results of the torque measurements and cure rate index—the differences between the individual results are not that noticeably large. The obtained results also prove the possibility of cross-linking of chloroprene rubber with silver(I) oxide, and its amount influences the vulcanization of the material.

### 3.2. Equilibrium Swelling of CR Cross-Linked with Silver(I) Oxide

The results obtained from equilibrium volume swelling confirmed the conclusions of the analysis of the vulcametric kinetics that the presence of silver(I) oxide causes the cross-linking of chloroprene rubber. The highest value of equilibrium volume swelling in toluene was obtained for the vulcanizate containing 1 phr of Ag_2_O (Q_V_^T^ = 20.44 cm^3^/cm^3^) (Table 3).

A much lower value of the equilibrium swelling in toluene was obtained for the vulcanizate containing 2 phr of Ag_2_O (*Q_V_*^T^ = 7.52 cm^3^/cm^3^). In turn, the CR vulcanizate containing 5 phr of Ag_2_O was characterized by the lowest *Q_V_*^T^ value, equal to 4.15 cm^3^/cm^3^. The values of the equilibrium volume swelling in heptane (*Q_V_*^H^) for all tested vulcanizates were comparable. The highest *Q_V_*^H^ value was obtained for the vulcanizates containing 1 or 2 phr of Ag_2_O, equal to 0.39 cm^3^/cm^3^. In turn, the highest value of volume equilibrium swelling in heptane was obtained for the vulcanizate containing 5 phr of Ag_2_O (*Q_V_*^H^ = 0.33 cm^3^/cm^3^). The value of negative equilibrium weight swelling in toluene (−*Q_W_*^T^) was the highest for the vulcanizate containing 1 phr of Ag_2_O (−*Q_W_*^T^ = 0.43 mg/mg), and the lowest for the vulcanizate containing 5 phr of Ag_2_O (−*Q_W_*^T^ = 0.13 mg/mg). In the case of negative equilibrium weight swelling in heptane (−*Q_W_*^H^), the values for all vulcanizates were almost identical and ranged between 0.05 and 0.07 mg/mg. The rubber volume fraction in toluene (*V_r_*^T^) increased with increasing Ag_2_O content in the vulcanizate. The lowest value of the rubber volume fraction in toluene was obtained for the vulcanizate containing 1 phr of Ag_2_O (*V_r_*^T^ = 0.047), and the highest value was obtained for the vulcanizate containing 5 phr of Ag_2_O (*V_r_*^T^ = 0.194). In the case of the rubber volume fraction in heptane (*V_r_*^H^), the values for all vulcanizates were comparable and ranged between 0.720 and 0.746. The cross-link density (*ν_e_*), calculated based on the interactions between chloroprene rubber and toluene, increased with the increasing content of silver(I) oxide in the vulcanizate. The lowest value of cross-linking density was obtained for the vulcanizate containing 1 phr of Ag_2_O (*ν_e_* = 0.24⋅10^−4^ mol/cm^3^), and the highest value was obtained for the vulcanizate containing 5 phr of Ag_2_O (*ν_e_* = 3.24⋅10^−4^ mol/cm^3^).

**Figure 2:**
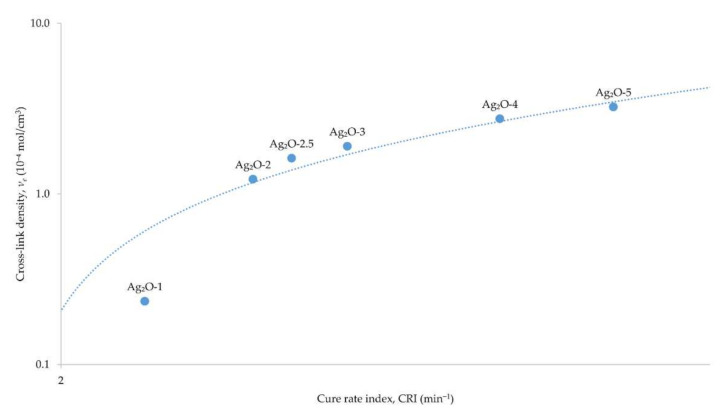
Dependence of the cross-link density on the cure rate index of the CR compositions containing silver(I) oxide (1–5 phr of Ag_2_O) on a logarithmic scale.

The obtained results for the equilibrium swelling of the vulcanizates confirm the ability to cross-link chloroprene rubber with silver(I) oxide. As the content of silver(I) oxide in the composition increases, the value of the cross-link density also increases. The *ν_e_* values show that 1 phr of Ag_2_O causes the creation of a less extensive network. The incorporation of at least 2 phr of Ag_2_O causes the formation of an appropriate network. Such dependence can be observed by the large increase in the cross-link density value between the compositions containing 1 and 2 phr of Ag_2_O. The increasing content of Ag_2_O in the composition causes the formation of an increasingly extensive network. Furthermore, a large increase in the cross-link density value can be observed with an increase in the Ag_2_O content in the composition from 3 to 4 phr. The cross-linking of CR with Ag_2_O probably takes place through the reaction of chlorine atoms with silver(I) oxide, as a result of which Lewis acid is generated in situ in the form of AgCl. The Lewis acid catalyzes the bonding of CR chains. The cross-linking according to this mechanism occurs when other metal oxides are used, such as the standardly used zinc oxide [35] or copper oxides [13,15]. There is also a noticeably close to linear relationship between the cross-link density and the cure rate index (Figure 2).

This confirms that, along with the increasing CRI values, the *ν_e_* values also increase. It is also important that as the slope of the curve increases, the effectiveness of using the cross-linking agent to form the network also increases. Moreover, the results obtained do not differ significantly from the relationship, and the CR composition containing 2.5 phr of Ag_2_O shows the greatest deviation towards greater cross-linking. The decreasing values of the negative equilibrium weight swelling in toluene with increasing content of silver(I) oxide in the vulcanizate indicate increasing resistance of the material to the solvent. The increasing value of the rubber volume fraction in toluene with the increase of Ag_2_O content confirms the conclusions drawn from the cross-link density calculations. The greater amount of Ag_2_O results in better cross-linking of CR, which translates into better resistance of the vulcanizate to the solvent. The equilibrium swelling values in heptane are noticeably lower than those determined in toluene. This is related to the polarity of the solvent and the rubber—nonpolar heptane does not affect the polar chloroprene rubber. This is evidenced by small amounts of leached substances from the samples (−*Q_W_*^H^) and the volume fraction of rubber in the samples (*V_R_*^H^) that were treated with heptane. In addition, the *Q_V_*^H^ values also decrease as the silver(I) oxide content in the composition increases.

### 3.3. Elasticity Constants of CR Cross-Linked with Silver(I) Oxide

The elasticity constants calculated from the Mooney-Rivlin equation allow the determination of the network formed during the cross-linking. The first elasticity constant (*C*_1_) is related to the degree of cross-linking—the greater the *C*_1_ value, the greater the vulcanizate degree of cross-linking is. The second elasticity constant (*C*_2_) can be equated with the deviation of the obtained network from the ideal network. The lowest value of the first elasticity constant was obtained for the vulcanizate containing 1 phr of Ag_2_O (*C*_1_ = 0.57 kG/cm^2^) and the highest value of the first elasticity constant was obtained for the vulcanizate containing 5 phr of Ag_2_O (*C*_1_ = 1.56 kG/cm^2^) (Table 4).

In the case of the results of the second elasticity constant, the smallest value was obtained for the vulcanizate containing 1 phr of Ag_2_O (*C*_2_ = 0.67 kG/cm^2^) and the highest value was obtained for the vulcanizate containing 3 phr of Ag_2_O (*C*_2_ = 2.92 kG/cm^2^). The obtained results confirm the observations made during the tests of vulcametric parameters and equilibrium swelling. The first elasticity constant values increase with increasing silver(I) oxide content in the composition, which is evidence of the increasing degree of cross-linking. The value of the first elasticity constant for the sample containing 1 phr of Ag_2_O does not differ significantly from the value for the sample containing 2 phr of Ag_2_O (for which *C*_1_ = 0.82 kG/cm^2^). This difference is not as significant as in the case of cross-link density calculations. This may be due to the structure of the network formed when 1 phr of Ag_2_O is used. The results of the second elasticity constant show that when the smaller amounts of silver(I) oxide are used, the values do not exceed 1 kG/cm^2^. In turn, for the vulcanizates containing at least 3 phr of Ag_2_O, the values are above 2 kG/cm^2^. Such results may prove that despite a smaller network expansion for vulcanizates with a smaller amount of silver(I) oxide, they create a more correct structure. Vulcanizates with more Ag_2_O are likely to form spherically hindered structures that make it difficult to form an ideal network, despite the high degree of cross-linking.

### 3.4. Real Extract of CR Cross-Linked with Silver(I) Oxide

To confirm that the cross-linking of chloroprene rubber with silver(I) oxide leads to network formation, the cross-linked samples were subjected to exhaustive extraction in the vapor of boiling acetone, which elutes non-rubber components and the gel fraction of CR. The lowest real extract value was obtained for the vulcanizate containing 1 phr of Ag_2_O (*E*_R_ = 0.043 mg/mg), and the highest value was obtained for the vulcanizate containing 5 phr of Ag_2_O (*E*_R_ = 0.089 mg/mg) (Table 4). The value of the real extract should be smaller, the greater the degree of cross-linking is. However, by correlating the results of the real extract with vulcametric parameters, equilibrium swelling measurements and elasticity constants, the relationship is reversed. The results obtained may prove that the entire cross-linking substance does not react during vulcanization. As the content of silver(I) oxide in the CR composition increases, more of it may not participate in the cross-linking of the chloroprene rubber and may be washed away by the solvent.

### 3.5. Mechanical Properties of CR Cross-Linked with Silver(I) Oxide

Mechanical strength tests have shown that the amount of silver(I) oxide affects the mechanical properties of vulcanizates. The stress at elongation 100% (*S*_e100_) for all vulcanizates was comparable and ranged between 1.16 and 1.32 MPa (Table 5).

**Figure 3:**
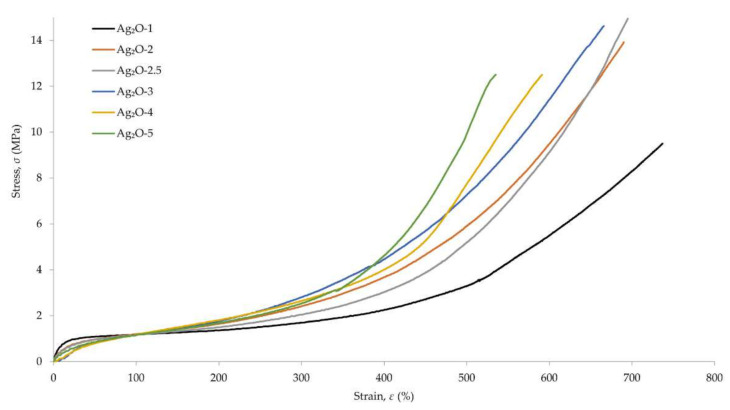
Dependence of the stress on the strain of CR cross-linked with silver(I) oxide (1–5 phr of Ag_2_O); T = 160 °C, *t* = 30 min.

The discrepancy of the stress at elongation 200% (*S*_e200_) results was greater, where the highest value was obtained for the vulcanizate containing 4 phr of Ag_2_O (*S*_e200_ = 2.02 MPa), and the lowest value was obtained for the vulcanizate containing 1 phr of Ag_2_O (*S*_e200_ = 1.36 MPa). The same relationship was observed for the stress at elongation 300% (*S*_e300_). The highest *S*_e300_ value was obtained for the vulcanizate containing 4 phr of Ag_2_O (*S*_e300_ = 3.17 MPa), and the lowest value was obtained for the vulcanizate containing 1 phr of Ag_2_O (*S*_e300_ = 1.72 MPa). The vulcanizate containing 1 phr of Ag_2_O was characterized by the lowest value of tensile strength (*TS*_b_), equal to 9.4 MPa. The incorporation of at least 2 phr of Ag_2_O into the composition resulted in an increase of TS_b_ values above 12 MPa. The highest *TS*_b_ value was obtained for the vulcanizate containing 2.5 phr of Ag_2_O, equal to 14.9 MPa. The longest elongation at break (*E*_b_) was obtained for the vulcanizate containing 1 phr of Ag_2_O, the value of which was 774%. The incorporation of at least 2 phr of Ag_2_O reduced the elongation at break to values below 700%. The shortest elongation at break was obtained for the vulcanizate containing 5 phr of Ag_2_O (*E*_b_ = 518%). Stress-strain changes of vulcanizates depending on the amount of Ag_2_O incorporated are shown in Figure 3.

**Figure 4:**
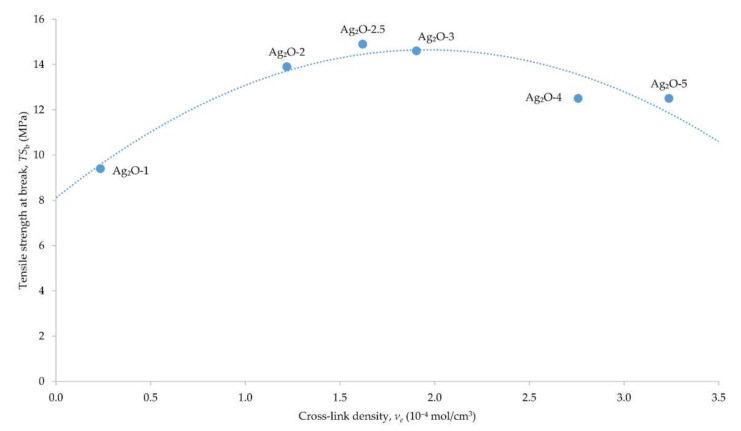
Tensile strength at break as a function of the cross-link density of the chloroprene rubber composition cured with silver(I) oxide (1–5 phr of Ag_2_O); T = 160 °C, *t* = 30 min.

The results obtained show that the degree of cross-linking influences the mechanical properties of the vulcanizates. Both the tensile strength and the elongation at break change noticeably when at least 2 phr of silver(I) oxide is used. The elongation at break values confirms the previous analyses of the degree of cross-linking—the increase in Ag_2_O content results in the greater degree of cross-linking of the vulcanizate. This is evidenced by the decreasing values of *E*_b_. Stress at elongation 100, 200 and 300%, which increases with the increase of Ag_2_O content in vulcanizates, proves the increasing stiffness of the obtained compositions. A greater amount of Ag_2_O increases the CR cross-link density, which also translates into stiffening of the material. These results correlate with the values of elongation at break. In the case of tensile strength, the highest TS_b_ value was obtained for the vulcanizate containing 2.5 phr Ag_2_O (Figure 4).

When larger amounts of Ag_2_O are incorporated, the tensile strength decreases. This is consistent with the dependence insofar that the tensile strength is not linearly related to the cross-link density [36]. After a certain cross-link density is obtained, the tensile strength is the highest and then decreases with increasing cross-linking of the material. In the case of the composition of CR/Ag_2_O, the highest tensile strength value was obtained for the vulcanizate containing 2.5 phr of Ag_2_O, with the cross-link density of 1.62⋅10^−4^ mol/cm^3^. By analyzing the influence of the surface properties of Ag_2_O on the properties of vulcanizates, it would be possible to conclude that both the small specific surface area (0.382 m^2^/g) and the large particle size (270 nm) will result in ineffective use of Ag_2_O as a CR cross-linking agent. However, the obtained results show, most importantly, that Ag_2_O is capable of cross-linking CR, and the vulcanizates are characterized by good tensile strength. This may indicate significant Ag_2_O reactivity, which eliminates the negative effects related to the size and surface of Ag_2_O.

### 3.6. Thermal Analysis of CR Cross-Linked with Silver(I) Oxide

**Figure 5:**
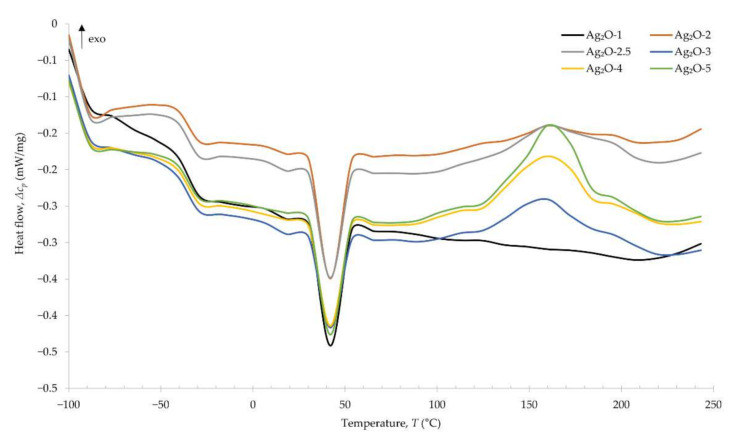
DSC curves of CR compositions containing silver(I) oxide (1–5 phr of Ag_2_O).

The use of differential scanning calorimetry allowed us to determine the glass transition temperature of vulcanizates. The values of the glass transition temperatures (*T*_g_) obtained for the individual compositions are presented in Table 6. For comparison, the glass transition temperature for standard cross-linked CR ranges from −50 to −40 °C [37,38]. A slight increase in the glass transition temperature of the tested compositions indicates the influence of silver(I) oxide on this parameter. However, the amount of Ag_2_O does not have a noticeable effect on the change in the glass transition temperature. At a temperature of 38–40 °C, a strong endothermic peak appears, indicating the melting of the crystalline structures formed during the storage of chloroprene rubber compositions. The emerging exothermic peak in the temperature range 158–164 °C indicates the cross-linking of CR with Ag_2_O. The values of the cross-linking temperatures (*T*_V_) obtained, cross-linking temperature ranges and cross-linking enthalpy (Δ*H*) for individual compositions are presented in Table 6. This confirms the observations from the determination of the degree of cross-linking—the use of 1 phr of Ag_2_O is insufficient to create an extensive network. Exclusively, the incorporation of at least 2 phr of Ag_2_O allows the CR to be effectively cross-linked. The higher amount of silver(I) oxide causes more effective cross-linking of the chloroprene rubber, as evidenced by the increasing amounts of energy released during this process (Figure 5).

**Figure 6:**
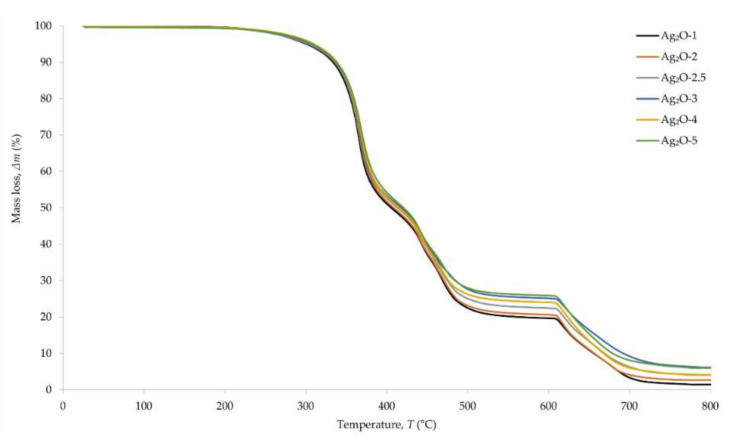
TGA curves of CR cross-linked with silver(I) oxide (1–5 phr of Ag_2_O); T = 160 °C, *t* = 30 min.

**Figure 7:**
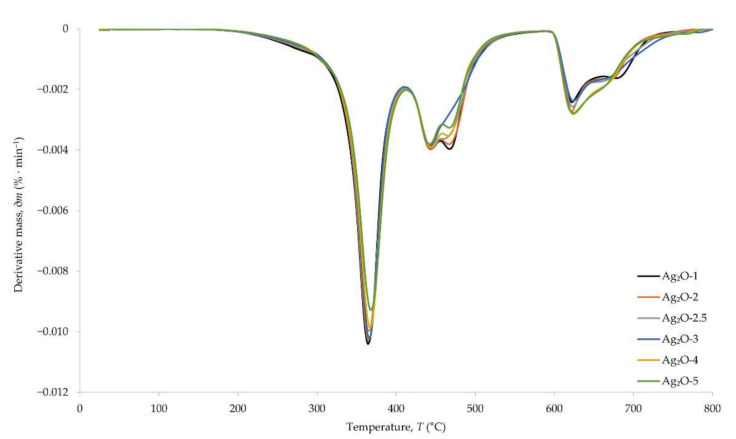
DTG curves of CR cross-linked with silver(I) oxide (1–5 phr of Ag_2_O); T = 160 °C, *t* = 30 min.

Thermographic curves allowed us to determine the processes taking place during the heating of CR compositions (Figure 6). The DTG curve, which is a derivative of the TGA curve, shows the change in the rate of decomposition of vulcanizates with increasing temperature (Figure 7). In the temperature range of 382–385 °C, the first weight loss of the samples appears, corresponding to the first stage of the pyrolysis of the composition. It reduces the mass of the samples to the value of 49–53% of the initial weight. The second stage of pyrolysis takes place at the temperature of ~470 °C, causing loss of the sample by another 24–27% of the initial weight. The last weight loss, which is 18–20% of the initial weight of the samples, occurs at the temperature of ~650 °C and corresponds to the combustion of soot formed during the pyrolysis. The burning of soot results from the change of gas from argon to air. The course of the thermographic curves does not differ significantly as a result of incorporating different amounts of silver(I) oxide into the CR composition. The differences can be seen in the amounts of material remaining after the second stage of pyrolysis and the subsequent burning of the soot. The greater amount of residue for samples containing more Ag_2_O means that the oxide is not susceptible to such high temperatures. Only rubber and stearic acid are thermally decomposed.

## 4. Conclusions

Summing up, the obtained results clearly indicate that chloroprene rubber can be cross-linked with silver(I) oxide, which is the most important novel insight of our research. This is evidenced by the measurements of vulcametric parameters, especially the torque increment after 30 min of heating the composition. The results of the equilibrium swelling measurements and the cross-link density calculations confirm the cross-linking ability of CR with Ag_2_O, which is an important novel finding. The degree of cross-linking of the vulcanizates increases with increasing Ag_2_O content of CR in the composition. The use of 1 phr of Ag_2_O allows the CR to cross-link; however, the resulting network is not sufficiently developed. By contrast, when at least 2 phr of Ag_2_O is used, the network formed is suitably developed. The results of the elasticity constants show that despite the insufficient development of the network with the use of 1 phr of Ag_2_O, a smaller amount of incorporated Ag_2_O allows the formation of a network with the correct structure. The real extract results may indicate incomplete utilization of larger amounts of Ag_2_O in the formation of the network. An important property of the obtained vulcanizates is their very good tensile strength. The presented results are better than those for the CR vulcanizates obtained in the standard method. Thermal analysis confirms that increasing the Ag_2_O content in the composition results in more efficient CR cross-linking. By contrast, the amount of Ag_2_O in the vulcanizate does not significantly affect the course of processes occurring in the material during its heating. The optimal condition is the use of 2.5 phr of Ag_2_O as CR cross-linking agent. This is evidenced by the highest tensile strength value. The use of more than 3 phr of Ag_2_O results in an increase in the cross-link density, with a simultaneous decrease of the tensile strength of the vulcanizates. Relative to the standard CR cross-linking agents, the new cross-linking agent Ag_2_O can be used in smaller amounts than ZnO, and moreover, it is not necessary to use MgO. As a result, it will be possible to reduce the production costs of such a composition, affecting the economic aspect.

## 5. Patents

The results presented in the article were included in the Polish Patent Declaration P.435392 of 22 September 2020.

## Figures and Tables

**Table 1:** Tested compositions and their designations.

CR (phr)	Ag_2_O (phr)	Stearic Acid (phr)	Symbol
100	1	1	Ag_2_O-1
100	2	1	Ag_2_O-2
100	2.5	1	Ag_2_O-2.5
100	3	1	Ag_2_O-3
100	4	1	Ag_2_O-4
100	5	1	Ag_2_O-5

**Table 2:** Vulcametric parameters of CR compositions; T = 160 °C.

Symbol	*t*_02_ (min)	*t*_90_ (min)	*M*_min_ (dN⋅m)	∆*M*_30_ (dN⋅m)	CRI (min^−1^)
Ag_2_O-1	6.93	53.36	0.89	2.16	2.15
Ag_2_O-2	1.18	43.55	1.05	3.03	2.36
Ag_2_O-2.5	0.85	41.91	1.15	3.55	2.44
Ag_2_O-3	0.71	39.70	1.16	3.95	2.56
Ag_2_O-4	0.50	34.72	1.46	4.68	2.92
Ag_2_O-5	0.45	31.55	1.45	4.99	3.22

Where: *t*_02_—scorch time; *t*_90_—vulcanization time; *M*_min_—minimum torque; Δ*M*_30_—torque increment after 30 min of heating; CRI—cure rate index.

**Table 3:** Equilibrium swelling of CR vulcanizates; T = 160°C, *t* = 30 min.

Symbol	*Q_V_*^T^ (cm^3^/cm^3^)	*Q_V_*^H^ (cm^3^/cm^3^)	*−Q_W_*^T^ (mg/mg)	*−Q_W_*^H^ (mg/mg)	*V_r_* ^T^	*V_r_* ^H^	*α_c_*	*ν_e_* (10^−4^ mol/cm^3^)
Ag_2_O-1	20.44 ± 0.53	0.39 ± 0.04	0.43 ± 0.02	0.05 ± 0.01	0.047 ± 0.001	0.722 ± 0.021	0.049	0.24
Ag_2_O-2	7.52 ± 0.16	0.39 ± 0.06	0.24 ± 0.03	0.06 ± 0.01	0.117 ± 0.002	0.720 ± 0.030	0.133	1.22
Ag_2_O-2.5	6.34 ± 0.22	0.37 ± 0.06	0.20 ± 0.01	0.06 ± 0.01	0.136 ± 0.004	0.732 ± 0.032	0.158	1.62
Ag_2_O-3	5.77 ± 0.05	0.36 ± 0.03	0.23 ± 0.01	0.06 ± 0.01	0.148 ± 0.001	0.733 ± 0.017	0.173	1.90
Ag_2_O-4	4.59 ± 0.11	0.35 ± 0.10	0.15 ± 0.01	0.07 ± 0.02	0.179 ± 0.003	0.746 ± 0.052	0.218	2.76
Ag_2_O-5	4.15 ± 0.02	0.33 ± 0.01	0.13 ± 0.01	0.05 ± 0.01	0.194 ± 0.001	0.736 ± 0.012	0.241	3.24

Where: *Q_V_*^T^, *Q_V_*^H^—equilibrium volume swelling in toluene or heptane, respectively; −*Q_W_*^T^, −*Q_W_*^H^—negative equilibrium weight swelling in toluene or heptane, respectively; *V_r_*^T^, *V_r_*^H^—rubber volume fraction in toluene or heptane, respectively; *α_c_*—degree of cross-linking; *ν_e_*—cross-link density.

**Table 4:** Elasticity constants and real extract of CR vulcanizates; T = 160 °C, *t* = 30 min.

Symbol	*C*_1_ (kG/cm^2^)	*C*_2_ (kG/cm^2^)	*E*_R_ (mg/mg)
Ag_2_O-1	0.57	0.67	0.043
Ag_2_O-2	0.82	0.92	0.052
Ag_2_O-2.5	0.98	0.72	0.050
Ag_2_O-3	1.10	2.92	0.060
Ag_2_O-4	1.46	2.89	0.081
Ag_2_O-5	1.56	2.37	0.089

Where: *C*_1_, *C*_2_—first or second elasticity constant, respectively; *E*_R_—real extract.

**Table 5:** Mechanical properties of CR vulcanizates; T = 160°C, *t* = 30 min.

Symbol	*S*_e100_ (MPa)	*S*_e200_ (MPa)	*S*_e300_ (MPa)	*TS*_b_ (MPa)	*E*_b_ (%)
Ag_2_O-1	1.16 ± 0.02	1.36 ± 0.01	1.72 ± 0.07	9.40 ± 0.48	774 ± 56
Ag_2_O-2	1.25 ± 0.09	1.68 ± 0.14	2.37 ± 0.17	13.9 ± 1.4	690 ± 48
Ag_2_O-2.5	1.16 ± 0.03	1.48 ± 0.05	2.01 ± 0.08	14.9 ± 0.4	698 ± 10
Ag_2_O-3	1.21 ± 0.04	1.73 ± 0.12	2.59 ± 0.28	14.6 ± 1.5	697 ± 97
Ag_2_O-4	1.32 ± 0.09	2.02 ± 0.22	3.17 ± 0.44	12.5 ± 0.2	581 ± 97
Ag_2_O-5	1.19 ± 0.02	1.75 ± 0.05	2.65 ± 0.10	12.5 ± 1.3	518 ± 78

Where: *S*_e100_, *S*_e200_, *S*_e300_—stress at elongation 100%, 200% or 300%, respectively; *TS*_b_—tensile strength at break; *E*_b_—elongation at break.

**Table 6:** Temperature and enthalpy of cross-linking determined by DSC for the CR compositions containing silver(I) oxide (1–5 phr of Ag_2_O).

Symbol	*T*_g_ (°C)	*T*_V_ Range (°C)	*T*_V_ (°C)	Δ*H* (J/g)
Ag_2_O-1	−37.91	111–199	124	0.26
			180	0.51
Ag_2_O-2	−37.47	139–200	160	10
Ag_2_O-2.5	−37.83	124–211	161	20
Ag_2_O-3	−38.65	120–193	158	20
Ag_2_O-4	−38.11	123–191	161	29
Ag_2_O-5	−37.78	128–189	164	36

Where: *T*_g_—glass transition temperature; *T*_V_ range—cross-linking temperature range; *T*_V_—cross-linking temperature; Δ*H*—cross-linking enthalpy.

## Data Availability

Data sharing not applicable.
